# A theoretical entropy score as a single value to express inhibitor selectivity

**DOI:** 10.1186/1471-2105-12-94

**Published:** 2011-04-12

**Authors:** Joost CM Uitdehaag, Guido JR Zaman

**Affiliations:** 1 Merck Research Laboratories, Department of Molecular Pharmacology and DMPK PO Box 20, 5340 BH, Oss, The Netherlands

## Abstract

**Background:**

Designing maximally selective ligands that act on individual targets is the dominant paradigm in drug discovery. Poor selectivity can underlie toxicity and side effects in the clinic, and for this reason compound selectivity is increasingly monitored from very early on in the drug discovery process. To make sense of large amounts of profiling data, and to determine when a compound is sufficiently selective, there is a need for a proper quantitative measure of selectivity.

**Results:**

Here we propose a new theoretical entropy score that can be calculated from a set of IC_50 _data. In contrast to previous measures such as the 'selectivity score', Gini score, or partition index, the entropy score is non-arbitary, fully exploits IC_50 _data, and is not dependent on a reference enzyme. In addition, the entropy score gives the most robust values with data from different sources, because it is less sensitive to errors. We apply the new score to kinase and nuclear receptor profiling data, and to high-throughput screening data. In addition, through analyzing profiles of clinical compounds, we show quantitatively that a more selective kinase inhibitor is not necessarily more drug-like.

**Conclusions:**

For quantifying selectivity from panel profiling, a theoretical entropy score is the best method. It is valuable for studying the molecular mechanisms of selectivity, and to steer compound progression in drug discovery programs.

## Background

In recent years, the kinase field has developed the practice of monitoring inhibitor selectivity through profiling on panels of biochemical assays [1-7], and other fields are following this example [8,9]. Such profiling means that scientists are faced with increasing amounts of data that need to be distilled into human sense. It would be powerful to have a good single selectivity value for quantitatively steering the drug discovery process, for measuring progress of series within a program, for computational drug design [10-12], and for establishing when a compound is sufficiently selective. However, in contrast to, for instance, lipophilicity and potency, where values such as logP or binding constant (K_*d*_) are guiding, quantitative measures for selectivity are still under debate. Often graphic methods are used to give insight, for example dotting a kinome tree [13,14], heat maps [4,6], or a radius plot, but such methods only allow qualitative comparison of a limited set of compounds at a time.

**Figure 1:**
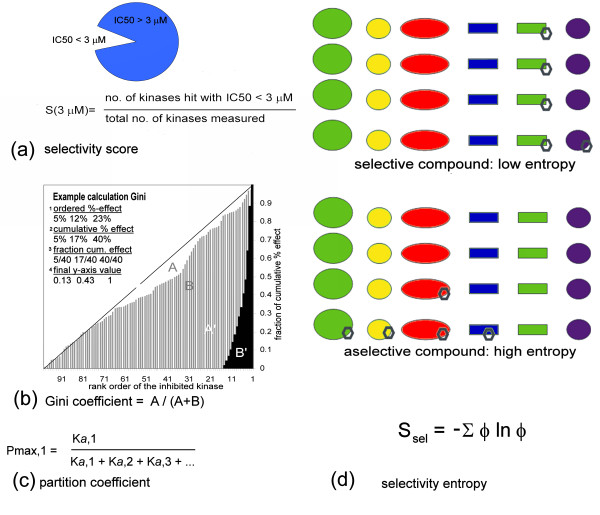
**Four ways to measure selectivity**. (a) The 'selectivity score' [5] is expressed as a fraction, as signified by the pie chart, and calculated by the formula given. (b) The four steps in calculation of the Gini coefficient [15] are indicated top-left inside the panel. For simplicity, a 3-protein example is used. The graph shows Gini scores from two inhibitor profiles on 100 kinases. The A'-profile is more specific. The area A' is larger than area A, and therefore the coefficient is larger. (c) The partition coefficient [16] is a ratio of association constants. The numbers 1, 2, 3... refer to kinases in the profiling panel. If *n *is a kinase number, then K_*a*, *n *_is defined as 1/K_*d*,*n*_. (d) The selectivity entropy. The various colors represent different proteins, and the hexagon a compound. Top: a selective compound binds almost exclusively to one protein, resulting in a narrow distribution across protein species. Bottom: a promiscuous compound binds to many different proteins, resulting in a broad distribution across protein species. The distribution can be quantified using Gibb's entropy definition (the formula shown).

To make quantitative selectivity comparisons, three notable methods have been proposed (Figure 1). The first is the 'selectivity score' [5], which simply divides the number of kinases hit at an arbitrary K_*d *_or IC_50 _value (e.g. 3 μM) by the number of kinases tested (S(3 μM), Figure 1a). A related score is S(10x), which divides the number of kinases hit at 10 times the K_*d *_of the target by the number of kinases tested [5]. The disadvantage of both methods is that 3 μM, or the factor 10, is an arbitrary cut-off value. For example, take two inhibitors, one that binds to two kinases with K_*d*_s of 1 nM and 1 μM, and another with K_*d*_s of 1 nM and 1 nM. Both are ranked equally specific by both S(3 μM) and S(10x), whereas the first compound is clearly more specific.

A less arbitrary parameter for selectivity is the Gini score [15]. This uses %-inhibition data at a single inhibitor concentration. These data are rank-ordered, summed and normalized (Figure 1b) to arrive at a cumulative fraction inhibition plot, after which the score is calculated by the relative area outside the curve (Figure 1b). Though this solves the problem with the selectivity score, it leaves other disadvantages. One is that the Gini score has no conceptual or thermodynamic meaning such as a K_*d *_value has. Another is that it performs suboptimally with smaller profiling panels [16]. In addition, the use of %-inhibition data makes the value more dependent on experimental conditions than a K_*d*_-based score [15]. For instance, profiling with 1 μM inhibitor concentration results in higher percentages inhibition than using 0.1 μM of inhibitor. The 1 μM test therefore yields a more promiscuous Gini value, requiring the arbitrary 1 μM to be mentioned when calculating Gini scores. The same goes for concentrations of ATP or other co-factors. This is confusing and limits comparisons across profiles.

A recently proposed method is the partition index [16]. This selects a reference kinase (usually the most potently hit one), and calculates the fraction of inhibitor molecules that would bind this kinase, in an imaginary pool of all panel kinases (Figure 1c). The partition index (P_max_) is a K_*d*_-based score with a thermodynamical underpinning, and performs well when test panels are smaller [16]. However, this score is still not ideal, since it doesn't characterize the complete inhibitor distribution in the imaginary kinase mixture, but just the fraction bound to the reference enzyme. Consider two inhibitors: A binds to 11 kinases, one with a K_*d *_of 1 nM and ten others at 10 nM. Inhibitor B binds to 2 kinases, both with K_*d*_s of 1 nM. The partition index would score both inhibitors as equally specific (P_max _= 0.5), whereas the second is intuitively more specific. Another downside is the necessary choice of a reference kinase. If an inhibitor is relevant in two projects, it can have two different P_max _values. Moreover, because the score is relative to a particular kinase, the error on the K_*d *_of this reference kinase dominates the error in the partition index. Ideally, in panel profiling, the errors on all K_*d*_s are equally weighted.

Here we propose a novel selectivity metric without these disadvantages. Our method is based on the principle that, when confronted with multiple kinases, inhibitor molecules will assume a Boltzmann distribution over the various targets (Figure 1d). The broadness of this distribution can be assessed through a theoretical entropy calculation (it is not actually measuring entropy). We show the advantages of this method and some applications. Because it can be used with any activity profiling dataset, it is a universal parameter for expressing selectivity.

## Results and discussion

### Theory

Imagine a theoretical mixture of all protein targets on which selectivity was assessed. No competing factors are present such as ATP. To this mixture we add a small amount of inhibitor, in such a way that approximately all inhibitor molecules are bound by targets, and no particular binding site gets saturated. A selective inhibitor will bind to one target almost exclusively and have a narrow distribution (low entropy, Figure 1d). A promiscuous inhibitor will bind to many targets and have a broad distribution (high entropy, Figure 1d). The broadness of the inhibitor distribution on the target mixture reflects the selectivity of the compound.

The binding of one inhibitor molecule to a particular protein can be seen as a thermodynamical state with an energy level determined by K_*d *_(through ΔG = RTlnK_*d*_). For simplicity we use the term K_*d *_to represent both K_*d *_and K_*i*_. The distribution of molecules over these energy states is given by the Boltzmann law. As the broadness of a Boltzmann distribution is measured by entropy, the selectivity implied in the distributions of Figure 1d can be captured in an entropy.

A similar insight is given by information theory. It is well-established that information can be quantified using entropy [17]. A selective kinase inhibitor can be seen as containing more information about which active site to bind than a promiscuous inhibitor. The selectivity difference between the inhibitors can therefore be quantified by information entropy.

The distribution of a compound across energy states is given by the Boltzmann formula [18]:(1)

Where ϕ_1 _is the fraction of molecules occupying state 1, and Δ*G*_1 _is the free energy of occupying state 1 when the inhibitor comes from solution. In order to arrive at a fraction, the denominator in equation (1) contains the summation of occupancies of all states, which are labelled *i*, with free energies Δ*G*_*i*_.

In general, entropy can be calculated from fractions of all *l *states using the Gibbs formula [18]:(2)

S_sel _is shorthand for selectivity entropy. Compared to the original Gibbs formulation, equation (2) contains a minus sign on the right hand to ensure that S_sel _is a positive value. Now, we need to evaluate equation (2) from a set of measurements. For this we need(3)

Where K_*a,i *_is the *association *constant of the inhibitor to target (or state) *i*, which is the inverse of the binding constant K_*d,i *_(which is a *dissociation *constant). In short: K_*a,i *_= 1/K_*d,i*_. If we express the free energy in units of 'per molecule' rather than 'per mole', equation (3) becomes(4)

and equation (1) can be rewritten as(5)

Using this result in equation (2) gives(6)

Simplifying notation gives(7)

Equation (7) defines how a selectivity entropy can be calculated from a collection of association constants K_*a*_. Here ΣK is the sum of all association constants.

It is most simple to apply equation (7) to directly measured binding constants or inhibition constants. Also IC_50_s can be used, but this is only really meaningful if they are related to K_*d*_. Fortunately, for kinases it is standard to measure IC_50 _values at [ATP] = K_M,ATP_. Ideally, such IC_50_s equal 2 times K_*d*_, according to the Cheng-Prusoff equation [19,20]. The factor 2 will drop out in equation (7), and we therefore can use data of the format IC_50_-at-K_M, ATP _directly as if they were K_*d*_.

### Protocol for calculating a selectivity entropy

From the above, it follows that a selectivity entropy can be quickly calculated from a set of profiling data with the following protocol:

1. Generate K_*a *_values by taking 1/K_*d *_or 1/IC_50_

2. Add all K_*a *_values to obtain ΣK

3. For every K_*a*_, calculate K_*a*_/ΣK

4. For every K_*a*_, evaluate (K_*a*_/ΣK) ln (K_*a*_/ΣK)

5. Sum all terms and multiply by -1

This process can be easily automated for use with large datasets [21] or internal databases.

Examples

The selectivity entropy is based on calculating the entropy of the hypothetical inhibitor distribution in a protein mixture. To give more insights into the properties of this metric, some examples are useful.

An inhibitor that only binds to a single kinase with a K_*d *_of 1 nM (K_*a *_= 10^9 ^M^-1^) has K_*a*_/ΣK_*a *_= 1. Then S_sel _= -[1 ln 1]= 0, which is the lowest possibly entropy.

An inhibitor that binds to two kinases (X and Y) with a K_*d *_of 1 nM has K_*x*_/ΣK_*a *_= K_*y*_/ΣK_*a *_= 0.5 and a selectivity entropy of -[0.5 ln 0.5 + 0.5 ln 0.5] = 0.69. Thus lower selectivity results in higher entropy.

If we modify the compound such that it still inhibits kinase X with a K_*d *_of 1 nM, but inhibits less strongly kinase Y with a K_*d *_of 1 μM, then the new inhibitor is more specific. Now K_*x*_/ΣK_*a *_= 10^9^/(10^9^+10^6^) and K_*y*_/ΣK_*a *_= 10^6^/(10^9^+10^6^), resulting in S_sel _= -[0.999 ln 0.999 + 0.001 ln 0.001] = 0.0079. This is less than 0.69. This shows that the selectivity entropy can distinguish in the case where the selectivity scores S(3 μM) and S(10x) cannot (see above).

A less selective inhibitor that binds three targets with K_*d*_s of 1 nM, has S_sel _= -3·[0.3 ln 0.3 ] = 1.08, and an even more promiscuous inhibitor that binds 5 targets, of which 3 at 1 nM, and 2 at 1 μM, has ΣK = 3·10^9^+ 2·10^6 ^= 3.002·10^9 ^and S_sel _= -3·[1·10^9^/3·10^9 ^ln 1·10^9^/3·10^9 ^] + 2·[1·10^6^/3·10^9 ^ln 1·10^6^/3·10^9 ^] = 3.07. Thus S_sel _gradually increases when more targets are more potently hit.

If we take the inhibitors A and B that were mentioned earlier, then A (with an inhibition profile of 1 nM, and ten times 10 nM), has ΣK = 1·10^9^+ 10·10^8 ^= 2·10^9 ^and S_sel _= - [1·10^9^/2·10^9 ^ln 1·10^9^/2·10^9 ^] + 10·[1·10^8^/2·10^9 ^ln 1·10^8^/2·10^9 ^] = 1.84. This is a more aselective value than inhibitor B with an inhibition profile of twice 1 nM, which has S_sel _= 0.69 (see above). Thus the selectivity entropy can distinguish in a case where the partition coefficient P_max _cannot.

### Comparison to other methods

Having defined the entropy, we next investigated its performance relative to the most widely-used methods, on a public profiling dataset of 38 inhibitors on 290 non-mutant kinases [5] (Table 1 and Additional file 1). The values for Gini score, S(3 μM), S(10x) and partition coefficient, were taken from earlier work [16]. To this we added a K_*a*_-Gini value and the selectivity entropy. The K_*a*_-Gini is a Gini score directly calculated on K_*a*_s, without reverting to %-inhibition values (see below). From each of these scores we determined an inhibitor selectivity ranking, and a rank order difference compared to the entropy method (Uitdehaag_S1). In addition, to get an overview of the profiling raw data [5], we appended an activity-based heat map (Uitdehaag_S1).

**Table 1:** Selectivity metrics calculated for the Ambit kinase profiling dataset

code	Gini	S3uM	S10x	Pmax	Ka-Gini	entropies
**PI-103**	0.41	0.00	0.00	0.97	0.99	0.05
**CI-1033**	0.72	0.15	0.00	0.88	0.99	0.17
**GW-2580**	0.54	0.01	0.00	0.92	0.99	0.26
**VX-745**	0.51	0.03	0.00	0.92	0.99	0.28
**Gefitinib**	0.65	0.07	0.00	0.89	0.99	0.44
**Lapatinib**	0.57	0.01	0.00	0.70	0.99	0.70
**Erlotinib**	0.75	0.15	0.00	0.67	0.99	0.88
**EKB-569**	0.78	0.18	0.00	0.67	0.99	0.89
**Imatinib**	0.79	0.07	0.02	0.65	0.99	1.05
**CP-724714**	0.49	0.02	0.01	0.33	0.99	1.10
**CP-690550**	0.66	0.03	0.01	0.50	0.99	1.11
**BIRB-796**	0.81	0.16	0.00	0.47	0.99	1.15
**PTK-787**	0.71	0.03	0.02	0.44	0.98	1.54
**SB-431542**	0.42	0.02	0.01	0.14	0.98	1.56
**MLN-518**	0.74	0.06	0.01	0.26	0.98	1.58
**LY-333531**	0.76	0.16	0.01	0.42	0.98	1.68
**CHIR-258**	0.76	0.33	0.01	0.44	0.97	1.80
**AZD1152HQPA**	0.78	0.10	0.02	0.34	0.98	1.91
**ABT-869**	0.82	0.16	0.01	0.32	0.98	1.93
**MLN-8054**	0.75	0.13	0.01	0.36	0.97	1.95
**Roscovitine**	0.33	0.03	0.03	0.12	0.98	2.02
**SU-14813**	0.70	0.51	0.02	0.18	0.97	2.04
**Sunitinib**	0.67	0.57	0.02	0.11	0.97	2.05
**GW-786034**	0.80	0.21	0.02	0.33	0.97	2.06
**SB-202190**	0.76	0.09	0.02	0.35	0.97	2.08
**Sorafenib**	0.82	0.18	0.10	0.46	0.97	2.15
**SB-203580**	0.77	0.10	0.02	0.24	0.97	2.26
**AMG-706**	0.82	0.09	0.04	0.22	0.97	2.36
**SNS-032**	0.80	0.13	0.06	0.30	0.97	2.44
**CHIR265**	0.74	0.13	0.12	0.36	0.96	2.47
**Flavopiridol**	0.77	0.19	0.17	0.42	0.95	2.50
**AST-487**	0.73	0.45	0.04	0.11	0.95	2.74
**ZD-6474**	0.79	0.27	0.26	0.28	0.94	2.88
**Staurosporine**	0.35	0.87	0.50	0.02	0.93	2.91
**VX-680**	0.76	0.38	0.03	0.15	0.93	3.09
**Dasatinib**	0.79	0.28	0.10	0.03	0.93	3.21
**PKC-412**	0.71	0.47	0.08	0.10	0.88	3.72
**JNJ-7706621**	0.74	0.37	0.08	0.10	0.87	3.73

Values for various selectivity metrics. In the Additional file 1, this table is extended with compound rank ordering and rank differences, and colour coded for clarity.

From the rankings it is apparent that each of the earlier methods such as the classic Gini score, S(3 μM) and S(10x) generate considerable ranking differences compared to all other methods. This was observed earlier [16]. For the Gini score, this is related to the conversion from IC_50 _to %-inhibition, because the K_*a*_-Gini gives more consistent rankings. For the S(3 μM) and the S(10x), the use of a cut-off is likely too coarse an approach. For instance in the case of S(10x), there are six inhibitors with a score of 0, making it impossible to distinguish between those highly specific compounds.

The newer methods such as P_max_, K_*a*_-Gini, and the selectivity entropy, give a more consistent ranking between them. For example, all three methods have PI-103, CI-1033, GW2580, VX-745 and gefitinib in their selectivity top five. There are differences however, most strikingly illustrated by the inhibitor SB-431542. This is ranked by P_max _as 31^st ^most selective, but by K_*a*_-Gini and the selectivity entropy as 15^th ^and 14^th ^(Uitdehaag_S1). Also S(3 μM) ranks this ALK5 inhibitor [22] as selective. However, SB-431542 hits four kinases with very similar IC_50_s between 100-300 nM, which leads to a broad partitioning over these kinases, resulting in a very promiscuous P_max _of 0.14. The partition coefficient therefore ranks SB-431542 as almost equally selective to sunitinib (P_max _= 0.11, rank 33). Nevertheless, sunitinib inhibits 181 kinases below 3 μM, and SB-431542 only 5. Therefore we think that K_*a*_-Gini and the selectivity entropy are a better 'general' measure of selectivity in this case.

Another inhibitor scored differently is MLN-518 [23], which ranks 26^st ^by P_max_, but 14^th ^and 15^th ^by K_*a*_-Gini and the selectivity entropy (Table 1 and Uitdehaag_S1). Again, these differences arise because this inhibitor hits 4 kinases with roughly equal potencies between 2-10 nM, leading to a promiscuous P_max _(0.26). However, MLN-518 only hits 10 kinases below 3 μM, making it intuitively more selective than e.g. ZD-6474 [24] (P_max _= 0.28, ranked 25^th ^by P_max_), which hits 79 kinases below 3 μM. These cases illustrate the earlier point that P_max _underscores inhibitors that only hit a few kinases at comparable potencies. The Gini score and selectivity entropy assign a higher selectivity to these cases.

Finally, any selectivity score should be in line with the visual ranking from a heat map. The Additional file 1 shows that, generally, compounds with a higher entropy indeed have a busier heat map. A few exceptions stand out, which by eye appear more promiscuous than their entropy ranking indicates, for instance SU-14813, sunitinib and staurosporin. However, these compounds have extreme low K_*d*_s on selected targets (SU-14813: 0.29 nM on PDGFRβ, sunitinib: 0.075 nM on PDGFRβ, staurosporin: 0.037 nM on LOK and 0.024 nM on SLK). Therefore they are relatively selective over activities in the 1-100 nM range, whereas these activities still fall within the highlighted ranges in Uitdehaag_S1. In a sense, the large dynamic range of the data limits visual assessment through a heat map.

### Consistency across profiling methods

**Figure 2:**
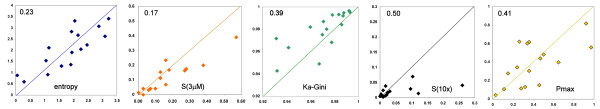
**Correlation between specificity values calculated from different datasets**. All x-axes: scores from binding data from the Ambit kinase dataset [5]. All y-axes: scores from activity data measured on the same compounds at Millipore. We calculated (in Microsoft Excel, for panels from left to right) the R-squares from linear regression as: 0.93, 0.92, 0.99, 0.54, 0.81 and the correlation coefficients as: 0.81, 0.90, 0.75, 0.57, 0.63. The straight line represents the ideal case of specificity values being insensitive to profiling method. The total squared distance of (normalized) data points to the straight line is given in the top left corner of each panel. For this latter calculation, data were normalized by dividing all values by the highest value in their set. Because the K_*a*_-Gini values are very unevenly distributed, the lowest value 0.93 was first subtracted from all data in this set. Irrespective of statistical method, the selectivity entropy, S(3 μM) and K_*a*_-Gini are the most robust metrics.

As a next step we selected 16 compounds from the public profile (Ambit) [5], and measured activity data on these using a different profiling service (Millipore, data available as Additional file 2). The 16 compounds represent a diversity of molecular scaffolds, promiscuity and target classes (Table 2). Also for these new data, we calculated the selectivity metrics (Uitdehaag_S2). In the ideal case, the selectivity values are similar irrespective of profiling technology (in the same way that a K_*d *_value is ideally independent of laboratory and assay format). The data of both methods are plotted in Figure 2.

**Table 2:** 16 compounds used to check the robustness of the selectivity metrics

code	name	structure
ABT-869 / AL-39324	linifanib	
AZD-1152HQPA	barasertib	
AZD-6474	vandetanib	
BAY43-9006	sorafenib	
BIRB-796	doramapimod	
BMS-345825	dasatinib	
CP-690550	tasocitinib	
MK-0457 / VX-680	tozasertib	
MLN-518 / CT-53518	tandutinib	
MLN-8054	-	
PI 103	-	
RAF-265 / CHIR-265	-	
SB 203580	-	
STI-571	imatinib	
SU-11248	sunitinib	
VX-745	-	

All metrics except the entropy and P_max _tend to be quite unevenly distributed. For instance all K_*a*_-Gini scores fall between 0.93 and 1.00, where they can theoretically range from 0 to 1. If we nevertheless calculate the correlation statistics between both datasets, the R-square from linear regression and the correlation indicate that the selectivity entropy, S(3 μM) and K_*a*_-Gini are the most robust methods (Figure 2).

It would be ideal if the absolute value of the metrics could also be compared between datasets. This means that a specificity of e.g. 1.2 in the first profile, would also score 1.2 in the second profile. To get insight in this, we calculated the best fit to a 1:1 correlation (the diagonal line in Figure 2), using normalized data. The K_*a*_-Gini score was rescaled to its useful range of 0.93-1.00 (see legend to Figure 2), and then fitted. The S(3 μM) and the selectivity entropy have the best fit. The fact that here the K_*a*_-Gini performs poorer is probably caused by the use of cumulative inhibition values (Figure 1b), which leads to the accumulation of errors (as pointed out in ref. 16).

In all fits, the P_max _and S(10x) scores show worse fits and more scatter, indicating that these methods generate more error in their final value. For S(10x) and for P_max_, this is because both methods make use of a reference value, usually the most potent IC_50_, and errors in this reference value propagate more than errors in other IC_50_s. Ideally, for S(10x) and P_max_, the reference value specifically would have to be more accurately established.

If all analyses are taken together, the selectivity entropy avoids many pitfalls of the other methods (see above), shows consistent compound ranking (Table 1, Uitdehaag_S1), and is among the most robust methods across profiling datasets (Figure 2). For this reason, we propose the entropy method as the best metric for general selectivity.

### Defining average selectivity

Quantification of selectivity helps to define when a compound is selective or promiscuous. Because of its consistency, the entropy method is ideally suited for benchmarking selectivity values. In the 290-kinase profiling dataset, the entropies are monomodally distributed, with an average of 1.8 (median of 1.9) and a standard deviation (σ) of 1.0 (not shown). Based on the correlation in Figure 2, it is expected that these statistics will be conserved in other profiling sets. Therefore, in general, a kinase compound with an entropy less than about 2 can be called selective, and more than 2 promiscuous. This provides a first quantitative definition of kinase selectivity.

### Selectivity of allosteric inhibitors

**Figure 3:**
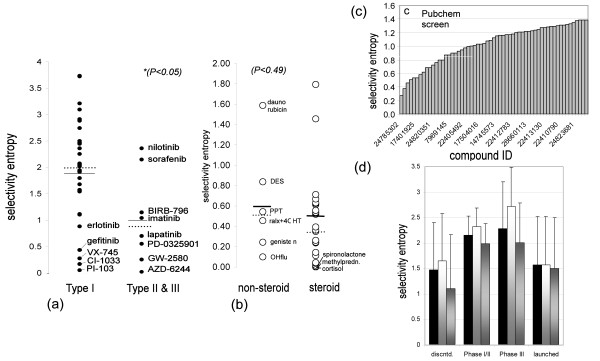
**Applications of selectivity entropy**. (a) Inhibitors that modify kinase conformation have higher selectivity. (b) Non-steroidal nuclear receptor antagonists are not more selective than steroidal antagonists. OHflu: hydroxyflutamide, ralx: raloxifene, 4OHT: 4-hydroxytamoxifen, PPT: propyl pyrazole triol, DES: diethylstilbestrol. Horizontal lines and dotted lines in panels (a) and (b) represent the average and median of each set, respectively. P values of two-tailed student-t tests are indicated. (c) Rank-ordering of hit selectivity in a panel of regulators of G-protein signalling. (d) Selectivity entropy of kinase inhibitors in clinical phase. Black bars: average entropy in that class. Light grey to white bars: averages restricted to oncology. Dark to light grey bars: averages for compounds of which the phase I trials were initiated before 2005. Error bars indicate one standard deviation. Numbers of datapoints used (column left to right): 10/8/6, 6/6/4, 6/4/4, 8/8/7. The discontinued class represent compounds that underwent clinical testing but were stopped. For the post-2005 projects, discontinued compounds have a lower entropy than the combined set of Phase III and launched compounds. As the number of non-oncology compounds is 2, 0, 2, 0 for each clinical bin respectively, this dataset only allows conclusions for the field of oncology.

It is generally thought that allosteric kinase inhibitors (known as type II, type III, or DFG-out inhibitors) are more selective [25,26]. The selectivity entropy now allows quantitative testing of this idea. We identified, from literature, which inhibitors in the profiling datasets are type II and III, based on X-ray structures. Sorafenib induces the kinase DFG-out conformation in B-RAF [27], nilotinib and gleevec in Abl [28], GW-2580 in Fms [29] and BIRB-796 in p38α [30]. Lapatinib induces a C-helix shift in EGFR [31]. PD-0325901 [32] and AZD-6244 induce a C-helix shift in MEK1 [32]. All other kinase inhibitors in the profile were labelled type I. Comparing the entropy distributions in both samples shows that type II/III inhibitors have significantly lower entropies (Figure 3a). Although other factors, such as the time at which a compound was developed, could influence the entropy differences, the correlation between low entropy and allostery strongly supports the focus on allostery for developing specific inhibitors [25,26].

Among the specific inhibitors in the type I category, 3D-structures of PI-103, CI-1033 and VX-745 bound to their targets have not been determined. Therefore, potentially, these inhibitors could also derive their specificity from a form of undiscovered induced fit. Indeed, VX-745-related compounds induce a peptide flip near Met109/Gly110 in P38α [33]. Of the five most selective compounds in Table 1, only gefitinib so far is undoubtedly a type I inhibitor [34], making this EGFR inhibitor an interesting model for the structural biology of non-allosteric specificity.

### Use of selectivity measures in nuclear receptor profiling

Selectivity profiling is most advanced in the kinase field, but is emerging in other fields. To illustrate that selectivity metrics such as the entropy can also be used with other target families, we investigated a long-standing question in the nuclear receptor field: are non-steroidal ligands more selective than steroidals? [35]. For this, we calculated the entropies of a published profile of 35 antagonists on a panel of 6 steroid receptors [9] (the androgen receptor, estrogen receptor α, estrogen receptor β, mineralocorticoid receptor, glucocorticoid receptor, and progesterone receptor). This shows that there are no statistically significant selectivity differences between steroidals and non-steroidals (Figure 3b). A more important determinant for selectivity could be, in parallel to kinase inhibitors, if a ligand induces a conformational change. Indeed, many nuclear receptor agonists are known to induce a transformation from a flexible receptor to a rigid agonistic form [36-40], or a heterodimer form [41,42]. In contrast, antagonists are know to displace helix 12 specifically from the agonistic form [36]. Thus, the large role of induced fit in ligand binding to nuclear receptors might explain the relative high selectivity of these ligands [9,36,43,44].

### Use in hit prioritization

Aside from solving questions in the structure-function area, the selectivity entropy can be used during drug discovery. Previously it has been shown that selectivity metrics can be used in lead optimization projects to classify compounds, set targets, and rationalize improvement [16]. In addition, metrics such as the entropy are useful in evaluating screening data, especially now screening larger compound collections in parallel assays is increasingly popular.

We downloaded PubChem data of 59 compounds tested in a panel of four assays for regulators of G protein signalling (RGS) [21]. These data were selected because they were publicly available and were neither a kinase nor a nuclear receptor panel. In addition the data were dose-response, were all in a similar assay format, and were ran in the same lab with the same compound set.

We calculated the compound entropies across the RGS panel, and used them for ranking, which immediately distinguishes the scaffolds that are specific (Figure 3c). The best are ID 24785302, a pyrazole-phenoxy derivative, and ID 24834029, a bicyclo-octane derivative, which are likely to be better lead optimization starting points than more promiscuous scaffolds. Triaging compounds by entropy is a far more time-efficient and unbiased way than manual evaluation of four parallel columns of data. Indeed, listing of the selectivity entropy in public databases of screening data would provide users with immediate information on scaffold promiscuity.

### Selectivity and clinical outcome

Finally, the selectivity entropy can be used to study clinical success. Selective compounds are generated because they are thought to be less toxic and therefore better doseable to effective ranges [45]. To test the hypothesis that clinically approved inhibitors are more selective, we binned the compounds in the public kinase profile [5] according to their clinical history, and calculated their average entropies (Figure 3d, Additional file 3). Compared to the average discontinued compound, the average marketed kinase inhibitor is not more selective, and the average Phase III compound is even significantly more aselective. To exclude therapy area effects, we also performed the analysis for compounds in the oncology area, which is the only therapeutic area with a statistically significant amount of projects. This leads to a similar conclusion (Figure 3d). To exclude effects of time from this analysis (more recently invented kinase inhibitors might be more selective, because of advances in the kinase field), we repeated the analysis for compounds that entered clinical phase I before 2005. This shows even more clearly that more succesful compounds are, if anything, more broadly selective (Figure 3d).

Behind such statistics lies the success of, for instance, the spectrum selective drugs dasatinib, sorafenib and sunitinib (an average entropy of 3.13), and the failure of the highly selective MEK-targeted drugs PD-0325901 and CI-1040 (an average entropy of 0.32). Because 66-100% of the analysed compounds in each clinical bin are (or were) developed for oncology, our conclusion is primarily valid for oncology, until more kinase inhibitors enter the clinic for other indications. Nevertheless, the finding that a selective kinase inhibitor has fewer chances of surviving early clinical trials fuels the notion that polypharmacology is sometimes required to achieve effect (in oncology) [45-47].

## Conclusions

In order to quantify compound selectivity as a single value, based on data from profiling in parallel assays, we have presented a selectivity entropy method, and compared this to other existing methods. The best method should avoid artifacts that obscure compound ranking, and show consistent values across profiling methods. Based on these criteria, the selectivity entropy is the best method.

A few cautionary notes are in order. First, the method is labelled an entropy in the sense of information theory [17], which is different to entropy in the sense of vibrational modes in enzyme active sites. Whereas these vibrations can form a physical basis for selectivity [39,48,49], our method is a computational metric to condense large datasets.

Secondly, any selectivity metric that produces a general value does not take into account the specific importance of individual targets. Therefore, the entropy is useful for generally characterizing tool compounds and drug candidates, but if particular targets need to be hit, or avoided, the K_*d*_s on these individual targets need to be monitored. It is possible to calculate an entropy on any particular panel of all-important targets, or to assign a weighing factor to every kinase, as suggested for P_max _[16] and calculate a weighted entropy. However, the practicality of this needs to be assessed.

Next, it is good custom to perform profiling in biochemical assays at [ATP] = K_M-ATP_, because this generates IC_50_s that are directly related to the ATP-independent K_*d *_value. However, in a cellular environment, there is a constant high (~5 mM) ATP concentration and therefore a biochemically selective inhibitor will act with different specificity in a cell. If the inhibitor has a specificity for a target with a K_M,ATP _above the panel average, then that inhibitor will act even more specifically in a cell and vice versa (K_M,ATP _values can generally be found on websites of profiling research organizations). Selectivity inside the cell is also determined by factors such as cellular penetration, compartimentalization and metabolic activity [39]. Therefore, selectivity from biochemical panel profiling is only a first step in developing selective inhibitors.

Another point is that any selectivity metric is always associated with the assay panel used, and the entropy value will change if an inhibited protein is added to the panel. Adding a protein that does not bind inhibitor will not affect the entropy value. In this way the discovery of new inhibitor targets by e.g. pulldown experiments, can change the idea of inhibitor selectivity, and also the entropy value. A good example is PI-103, the most selective inhibitor in Table 1, which in the literature is known as a dual PI3-kinase/mTOR inhibitor [50], and which appears specific in Table 1 because PI3-kinase is not incorporated in the profiling panel.

In addition, an inhibitor that hits 2 kinases at 1 nM from a panel of 10 has the same selectivity entropy as an inhibitor that inhibits 2 kinases at 1 nM in a panel of 100. However, intuitively, the second inhibitor is more specific (the 'selectivity score' differentiates in this case). This illustrates that it is important to compare entropy scores on similar panels. At the same time, when results from different panels are weighed, as in the example, it should not be assumed for the first inhibitor, that it is inactive against all 90 other kinases in the second panel. It would be better to assign an average K_*d *_where measurements are missing. In that case the first inhibitor would score a more promiscuous entropy compared to the second inhibitor.

Finally it must be stressed that the selectivity entropy could be applied in many more fields. It could, for instance, be a useful metric in the computational studies that attempt to link compound *in vitro *safety profiles to compound characteristics [51-53]. Currently, that field uses various forms of 'promiscuity scores' which bear similarity to the selectivity score. A more robust and non-arbitrary metric such as the selectivity entropy could be of help in building more detailed pharmacological models of compound activity-selectivity relationships [51-53].

In summary, the selectivity entropy is a very useful tool for making sense of large arrays of profiling data. We have demonstrated its use in characterizing tool compounds and drug candidates. Many more applications are imaginable in fields where an array of data is available and the selectivity of a response needs to be assessed. In that sense, the selectivity entropy is a general aid in the study of selectivity.

## Methods

### Calculation of other selectivity scores

For comparisons between currently used methods, we calculated the selectivity scores S(3 μM) and S(10x) as outlined above and in ref. 5. The partition coefficient P_max _was calculated as originally proposed [16], by taking the K_*a *_value of the most potently hit kinase, and dividing it by Σ K_*a*_. It is worth to note that the partition coefficient is the same as ϕ_l _in our entropy equation (eq. 2).

The Gini score was calculated from data on %-inhibition [15]. In Figure 1b, these data were extracted from K_*d *_values using the Hill expression: %-inhibition = 100/(1+10^-(pKd - pconc)^), where pK_*d *_= -log (K_*d*_) and pconc = -log (inhibitor concentration evaluated). In addition, to work more directly with K_*d*_s, we also introduce a K_*a*_-Gini score, in which association constants are used for rank-ordering the kinase profile. From this K_*a*_-rank ordering, a cumulative effect is calculated and normalized, after which the areas are determined, in the same way as for the original Gini score [15]. All calculations were done in Microsoft Excel.

### Sources of existing and new data

For our comparative rank-ordering (Table 1, Uitdehaag_S1) we used the publicly available dataset released by Ambit http://www.ambitbio.com, which contains binding data (K_*d*_s) of 38 inhibitors on 290 kinases (excluding mutants), and which is currently the largest single profiling set available [5].

For comparing profiles across methods (Figure 2), we selected 16 kinase inhibitors of the Ambit profile (Table 2) and submitted these to the kinase profiling service from Millipore (http://www.millipore.com/drugdiscovery/svp3/kpservices, data available as Additional file 2). Both profiling methods are described earlier [3,5,14] and differ (among other variations) in the following way: Ambit uses a competitive binding setup in absence of ATP on kinases from T7 or HEK293 expression systems [14]. Millipore uses a radioactive filter binding activity assay, with kinases purified from *Escherichia coli *or baculovirus expression systems [3]. All Millipore profiling was done on 222 human kinases at [ATP] = K_M,ATP_.

For comparing inhibitors with an allosteric (actually: induced fit) profile (Figure 3a), we used data from the Ambit profile [5], supplemented with Millipore profiling data on nilotinib, PD-0325901 and AZD6244, because these important inhibitors were lacking in the Ambit dataset (data available in Additional file 2).

For comparing nuclear receptor data (Figure 3b), we used the published profiling dataset of 35 inhibitors on a panel consisting of all six steroid hormone receptors [9] The data we used were EC_50_s in cell-based assays.

For evaluation of a screening dataset (Figure 3c), we selected data from the PubChem initiative, determined at the University of New Mexico on regulators of G protein signalling (isoforms 4, 19, 7 and 16. Assay identifiers: 1872, 1884, 1888 and 1869) [21].

For evaluating clinical success (Figure 3d), we tracked the clinical status of each compound in the Ambit profile using the Thompson Pharma^® ^database (status February 2011, analysis availabe as Additional file 3).

## Authors' contributions

JU conceived the entropy principle and drafted the manuscript. GZ organized the kinase profiling data and helped to draft the manuscript. All authors read and approved the final manuscript.

## Supplementary Material

Additional file 1 **Selectivity metrics, heat maps, and selectivity rank ordering for the Ambit profiling dataset (an extension of Table **1**)**. Click here for file

Additional file 2 **EC50 values and selectivity metrics from an activity based profiling of 16 reference inhibitors**. Click here for file

Additional file 3 **Selectivity entropy and status of clinically tested kinase inhibitors**. Click here for file
