# Supplementary figures and images for: A theoretical entropy score as a single value to express inhibitor selectivity
Source: BMC Bioinformatics. 2011 Apr 12;12:94. doi: 10.1186/1471-2105-12-94 (PMC3100252; doi:10.1186/1471-2105-12-94)

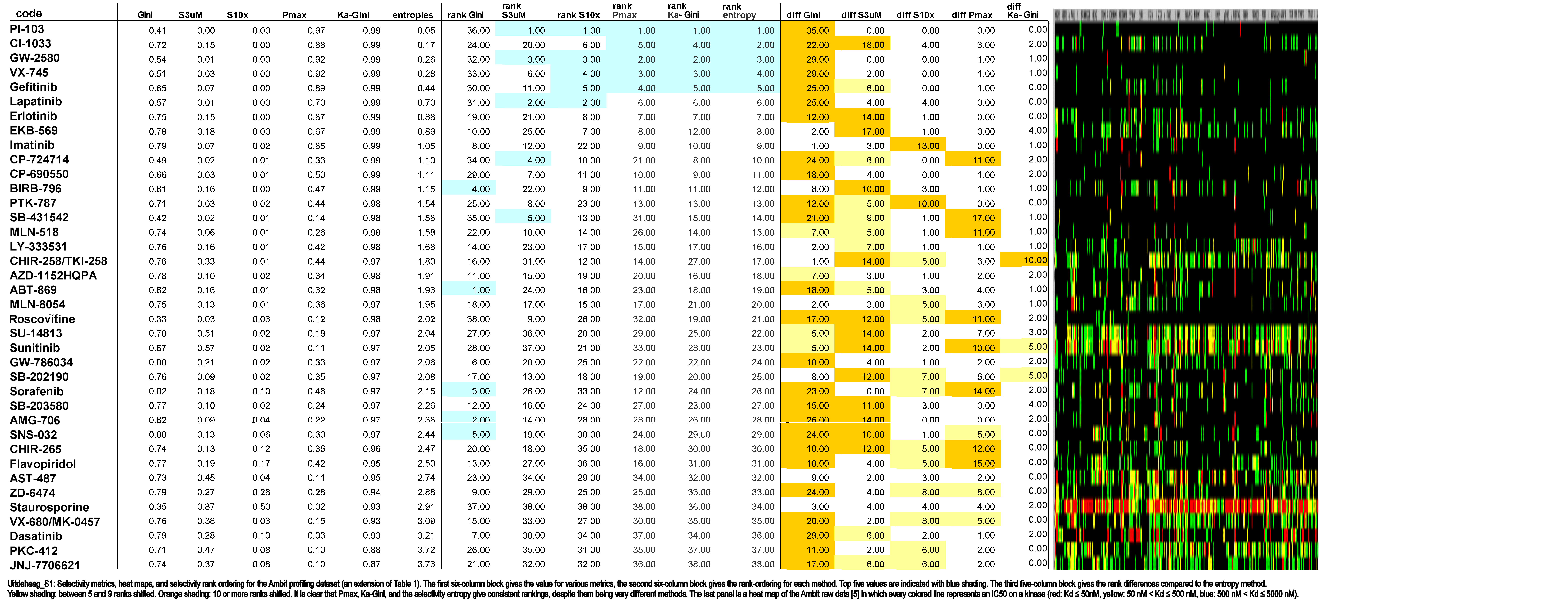

Supplement: Additional file 1 — Selectivity metrics, heat maps, and selectivity rank ordering for the Ambit profiling dataset (an extension of Table 1). [file 1471-2105-12-94-S1.TIFF]
