# Supplementary material for: A theoretical entropy score as a single value to express inhibitor selectivity
Source: BMC Bioinformatics. 2011 Apr 12;12:94. doi: 10.1186/1471-2105-12-94 (PMC3100252; doi:10.1186/1471-2105-12-94)
Supplement: Additional file 2 — EC50 values and selectivity metrics from an activity based profiling of 16 reference inhibitors. [file 1471-2105-12-94-S2.PDF]

Uitdehaag *et al*. additional data S2

inhibitors and potencies (expressed as pEC50 = -log EC50 (EC50 in M)) — allosteric inhibitors: AZD6244, PD-0325901

target	ABT-869	AZD-1152	AZD-6474	BIRB796	CP-690550	dasatinib	imatinib	MLN-518	MLN-8054	PI-103	RAF-265	SB-203580	sorafenib	sunitinib	VX-745	VX-680	AZD6244	PD-0325901	nilotinib
ABL1			6.63			> 8	7.56		6.26		6.99		5.63	5.31		7.49			> 8
ABL2			5.65			> 8	7.63		5.97		6.15		5.53			7.12			> 8
ACVR1B						5.96													
AKT1																			
AKT2																			
AKT3																			
ALK	6.07													6.18					
AURKA	6.19	7.62			5.27	5.13	< 4.52	< 4.52	> 8				5.19	6.01		> 8			
AXL	6.6								6.04				5.62	7.03		5.57			
BLK			6.67			> 8			6.11					6.78		5.99			
BMX			5.53			> 8			6.24							7.02			
BRSK1														5.61					
BRSK2														6.14					
BTK			6.14			> 8								4.78		5			
CAMK1	< 4.52													5.92					
CAMK1D	5.45													6.12					
CAMK2B														6.41					
CAMK2D					6.01									7.83					
CAMK2G					6.02									7.93					
CAMK4														5.59					
CDC2/CCNB1																			
CDC42BPA																			
CDC42BPB																			
CDK2/CCNA2																			
CDK2/CCNE1																			
CDK3/CCNE1																			
CDK5														5.4					
CDK5R1														5.32					
CDK7/CCNH/MNAT1														6.77					
CDK9/CCNT1																			
CHEK1														6.02					
CHEK2									5.06					7					
CHUK																			
CKI1												7.52		5.56					
CLK2														6.94					
CLK3									5.85										
CSF1R	> 8		5.19			> 8	6.93	7.56			6.89		7.95	> 8		5.63			7.26
CSK						7.93													5.82
CSNK1D												6.99		6.57					
CSNK1G1														5.54					
CSNK1G2														5.82					
CSNK1G3									5.29					5.92					
CSNK2A1																			
CSNK2A2																			
DAPK1																			
DAPK2														5.53					
DAPK3										< 4.52				5.06					
DCAMKL2																			
DDR2	6.88			6.79		7.25	6.99		5.58		6.7		7.6			5.96			7.86
DMPK																			
DYRK2																			
EGFR			7.29			6.44													
EPHA1			6.85			7.89			6.49		6.01	5.96	6.18			6.33			6.23
EPHA2	5.82		6.45	5.8		7.73	5.3		6.2		5.97		6.32			6.58			7.61
EPHA3	5.9		6.06			7.66			5.57				6.08	5.41		5.89			7.04
EPHA4			6.18			7.74			5.74				5.41			5.41			
EPHA5																			
EPHA7	6.86		5.83	6.85									6.18						
EPHA8	5.52		6.52	6.5		7.32	5.54				6.61		6.2			5.92			7.39
EPHB1	5.89		6.46			7.28			6.23				6.4	5.83		5.97			6.59
EPHB2	6.61		6.26			7.64			6.36				5.87	5.49		5.45			5.89
EPHB3						6.87													
EPHB4	5.77		6.43			7.74			6.74			5.09	6.01	5.75		6.21			
ERBB4		5.98	5.71			7.03													
FER									5.77		5.42			6.11					
FES														6.63					
FGFR1	6.52		6.28			5.66		5.46	6.59		6.87		6.71	6.22		6.38			
FGFR2	5.69		5.75			5.49		5.23	5.84		5.27		6.33	6.09		5.99			
FGFR3			5.88						6.01				5.88	6.16		5.79			
FGFR4																			
FGR			6.51			> 8			5.98					6.71		6.36			5.45
FLT1	> 8	5.96	7.29	6.06		5.41		5.44	5.59		6.72		> 8	7.7		6.52			
FLT3	> 8	6.72						7.05		5.49			7.47	> 8		7.13			
FLT4	> 8	6.51	6.96	5.55	5.34	5.25	5.62	6.14	6.6	4.73	6.52		7.9	> 8		6.62		5.1	
FRAP1										6.95									
FRAP1/FKBP1A										7.1									
FRK	6.95		6.08	6.01		> 8	6.05				6.49	5.74	7.03	6.13		5.39		5.44	7.52
FYN			6.39		5.6	> 8			6.11		5.78			6.21		6.22			5.51
GRK5																			
GRK6																			
GRK7																			
GSG2														6.49					
GSK3A																			
GSK3B												6.92							
HCK	5.48	6.29	6.62	5.24		> 8	5.59		6.99		7.1		5.9	7.07		6.12			7.22
HIPK1													5.49	5.49					
HIPK2	5.85												6.37	6.3					
HIPK3	5.9												6.38	5.86					
IGF1R	5.9													5.43		6			
IKBKB																			
INSR	4.86								4.74					5.92		6.22			
INSRR	5.69													5.82		5.52			
IRAK1														6.21					
IRAK4	5.57		6.08											5.91					
ITK														6.7		6.14			
JAK2					> 8	5.42								5.73		6.15			
JAK3					> 8														
KDR	> 8	6.21	7.1	5.63				5.31	5.73		6.86		7.86	7.76		5.94			
KIT	6.87		5.45			6.85	5.35	6.35					5.64	6.91					
LCK	6.32	5.01	7.37	5.87	5.6	> 8	6.47	5.11	5.85		5.28	5.68	6.02	7.16		6.59			< 4.52
LIMK1						6.4							6.1			6.6			
LYN	5.61		6.9			> 8	6.25		5.56		7.04		6.22	6.8		6.17			7.06
MAP2K1																	5.53	6.74	
MAP2K4																			
MAP2K6				6.41		5.77						6.15	6.22		6.21				6.41
MAP2K7		< 4.52																	
MAP3K5																			
MAP3K7				5.86							6.33		5.86	6.55					
MAP3K9	5.65		5.59			5.76					6.12			6.17		7.07			
MAP4K2	< 4.52					5.31					5.75			6.43					
MAPK1																			
MAPK10	5.3			5.79								6.64							5.7
MAPK11	6.51			7.08		6.79						7.21	7.19		7.3				7.3
MAPK12	5.79			6.67															
MAPK13				6.39															
MAPK14				4.67		6.44						7.05	7.02		6.44				< 4.52
MAPK3																			
MAPK8																			
MAPKAPK2																			
MAPKAPK3																			
MAPKAPK5																			
MARK2					5.73									6.12					
MELK														6.48		6.43			
MERTK	7.06		5.45						6.48				5.87	7.71		6.37			
MET	6.43															5.14			
MINK1				5.84							5.86			5.9					
MKNK2	6.46		5.44										6.64						
MST1R	5.7															5.03			
MUSK	7												< 4.52	< 4.52		5.9			< 4.52
MYLK														6.13					
NEK11						5.2													
NEK2											4.66								
NEK3																			
NEK6																			
NLK						6.1						5.76							
NTRK1	6.86	5.84		6.13				5.72	6.25		4.88		6.58	7.45		7.91			5.55
NTRK2	4.77	5.26		6.19				5.39	5.52		5.1		6.19	6.63		6.6			5.96
NUAK1					5.61									7.02		6.52			
PAK2																			
PAK3																			
PAK4																			
PAK6																			
PAK7														5.53					
PASK	5.87																		
PDGFRA	7.5	5.49				7.19		6.55					6.57	7.28					
PDGFRB	6.96					6.54		5.76					5.72	6.63					
PDPK1																			
PHKG2														7.1					
PIM1																			
PIM2																			
PIM3														4.82					
PKN2					6.12														
PLK1																			
PLK2	7.56																		
PLK3																			
PRKAA2														6.4					
PRKACA	5.27																		
PRKCA																			
PRKCB1																			
PRKCB2																			
PRKCD					5.87														
PRKCE																			
PRKCG																			
PRKCH																			
PRKCI																			
PRKCQ																			
PRKCZ																			
PRKD1										4.58				6.03					
PRKD2														5.53					
PRKG1A	6.14																		
PRKG1B	6.52																		
PRKX																			
PTK2	5.75								5.37					6.32					
PTK2B														6.16					5.77
PTK6	5.5		7.28			7.81						6.11		5.65		5.43			6.65
RAF1	5.72			6.23		6.76	6.34			5.67	7.25	6.66	7.35						7.05
RET	7.28	7.08	7.46	5.76		5.89					7.97		> 8	7.29		7.19		5.71	5.6
RIPK2			6.48			6.46						6.52	5.46						
ROCK1																			
ROCK2	6.88				5.96									5.62					
ROS1																			
RPS6KA1	6.73													6.92					
RPS6KA2	5.77								5.63					6.44		5.39			
RPS6KA3									5.43					5.97					
RPS6KA4	6.65													5.73					
RPS6KA5	7.03																		
RPS6KA6									5.89					6.58					
RPS6KB1	6.66												5.88	6.01					
SGK	5.57													4.95					
SGK2	6.04																		
SGK3	< 4.52																		
SNF1LK			5.43			> 8			5.73					5.58					
SRC			6.31			> 8			4.88		4.52		4.52	5.98		5.98			< 4.52
SRC(1-530)	6.39		7.28			> 8		< 4.52	5.71		5.63	5.46	6.34	6.83		6.2			6.47
SRPK1																			
SRPK2									5.71										
SRPK3	5.74																		
STK10			6.48	5.56							6.11		< 4.52	6.32					
STK17A									5.4					6.96					
STK24						5.43								5.9					
STK3																			
STK33	4.84												4.81	6.31					
STK4														6.33					
SYK						5.64					5.99					5.59			
TAOK1	7.04										6.46		6.31			5.91			5.75
TAOK2	5.98										> 8		7.12			6.21			5.2
TAOK3	6.83										7.37		7.54			5.71			
TBK1														5.83					
TEK	6.49	5.19	5.95	7.6					5.79				5.17			6.27			< 4.52
TLK2														5.79					
TNK2	4.92		5.28		5.43	> 8			5.22			4.92		5.3		5.68			
TSSK1B														6.37					
TSSK2																			
TXK			5.27			> 8						5.01							
TYRO3	5.4		6.37	5.52					5.5					5.96					
ULK2														6.5					
ULK3											5.88			6.35		5.63			
VRK2																			
WNK2										< 4.52									
WNK3	5.45					5.3													
YES1			7.38		5.45	> 8		6.1	6.66		6.31			7.23		7.16			
ZAP70																			
	ABT-869	AZD-1152	AZD-6474	BIRB796	CP-690550	dasatinib	imatinib	MLN-518	MLN-8054	PI-103	RAF-265	SB-203580	sorafenib	sunitinib	VX-745	VX-680	AZD6244	PD-0325901	nilotinib
selectivity entropy	2.834	1.366	3.066	2.117	0.915	3.407	1.536	1.300	1.903	0.876	2.245	2.047	2.668	3.319	0.590	2.625	0.020	0.550	2.360
selectivity score	0.235	0.041	0.163	0.068	0.041	0.176	0.041	0.032	0.154	0.014	0.113	0.041	0.167	0.389	0.000	0.199	0.000	0.005	0.095
Ka-Gini score	0.968	0.993	0.963	0.984	0.995	0.943	0.992	0.993	0.980	0.995	0.982	0.987	0.975	0.949	0.997	0.972	1.000	1.000	0.980
S10x	0.025	0.007	0.041	0.011	0.005	0.070	0.009	0.005	0.002	0.005	0.014	0.016	0.029	0.038	0.005	0.020	0.002	0.002	0.027
Pmax	0.132	0.603	0.117	0.440	0.481	0.041	0.399	0.621	0.617	0.561	0.304	0.342	0.149	0.107	0.820	0.278	0.999	0.857	0.208
