# Supplementary material for: A theoretical entropy score as a single value to express inhibitor selectivity
Source: BMC Bioinformatics. 2011 Apr 12;12:94. doi: 10.1186/1471-2105-12-94 (PMC3100252; doi:10.1186/1471-2105-12-94)
Supplement: Additional file 3 — Selectivity entropy and status of clinically tested kinase inhibitors. [file 1471-2105-12-94-S3.PDF]

Uitdehaag *et al*. additional file S3

compound code	clinical phase	first in man		entropy			
	(Thomson Pharma, Feb. 2011)			all	onc. only	pre-2005	non-onc.
**CI-1033**	**discontinued**	**2001**	**oncology**	0.17	0.17	0.17	
**VX-745**	**discontinued**	**2000**	**inflammation**	0.28		0.28	0.28
**EKB-569**	**discontinued**	**2003**	**oncology**	0.89	0.89	0.89	
**CP-724714**	**discontinued**	**2002**	**oncology**	1.10	1.10	1.10	
**BIRB-796**	**discontinued**	**2001**	**inflammation**	1.15		1.15	1.15
**PTK-787**	**discontinued**	**2005**	**oncology**	1.54	1.54		
**MLN-8054**	**discontinued**	**2005**	**oncology**	1.95	1.95		
**SU-14813**	**discontinued**	**2005**	**oncology**	2.04	2.04		
**SNS-032**	**discontinued**	**2006**	**oncology**	2.44	2.44		
**VX-680/MK-0457**	**discontinued**	**2004**	**oncology**	3.09	3.09	3.09	
			**average**	1.47	1.65	1.11	0.72
			st. dev.	*0.93*	*0.93*	*1.05*	*0.62*
**RAF-265**	**Phase I**	**2006**	**oncology**	2.47	2.47		
**MLN-518**	**Phase II**	**2002**	**oncology**	1.58	1.58	1.58	
**CHIR-258/TKI-258**	**Phase II**	**2004**	**oncology**	1.80	1.80	1.80	
**AZD-1152**	**Phase II**	**2005**	**oncology**	1.91	1.91		
**roscovitine**	**Phase II**	**2001**	**oncology**	2.02	2.02	2.02	
**Flavopiridol**	**Phase II**	**2004**	**oncology**	2.50	2.50	2.50	
			**average**	2.05	2.05	1.97	
			st.dev.	*0.37*	*0.37*	*0.39*	
**CP-690550**	**Phase III**	**2004**	**inflammation**	1.11		1.11	1.11
**LY-333531**	**Phase III**	**1998**	**diabetic retinopathy**	1.68		1.68	1.68
**ABT-869**	**Phase III**	**2006**	**oncology**	1.93	1.93		
**AMG-706**	**Phase III**	**2004**	**oncology**	2.36	2.36	2.36	
**ZD-6474**	**Phase III**	**2003**	**oncology**	2.88	2.88	2.88	
**PKC-412**	**Phase III**	**2005**	**oncology**	3.72	3.72		
			**average**	2.28	2.72	2.01	1.40
			st.dev.	*0.93*	*0.77*	*0.77*	
**Gefitinib**	**launched**	**2000**	**oncology**	0.44	0.44	0.44	
**Lapatinib**	**launched**	**2002**	**oncology**	0.70	0.70	0.70	
**Erlotinib**	**launched**	**2002**	**oncology**	0.88	0.88	0.88	
**Imatinib**	**launched**	**2000**	**oncology**	1.05	1.05	1.05	
**Sunitinib**	**launched**	**2002**	**oncology**	2.05	2.05	2.05	
**GW-786034**	**launched**	**2006**	**oncology**	2.06	2.06		
**Sorafenib**	**launched**	**2001**	**oncology**	2.15	2.15	2.15	
**Dasatinib**	**launched**	**2004**	**oncology**	3.21	3.21	3.21	
			**average**	1.57	1.57	1.50	
			st.dev.	*0.95*	*0.95*	*1.00*	
**GW-2580**	**tool**	**na**	**inflammation**	0.26			0.26
**SB-431542**	**tool**	**na**	**misc.**	1.56			1.56
**SB-202190**	**tool**	**na**	**inflammation**	2.08			2.08
**SB-203580**	**tool**	**na**	**inflammation**	2.26			2.26
**AST-487**	**tool**	**na**	**misc.**	2.74			2.74
**Staurosporine**	**tool**	**na**	**misc.**	2.91			2.91
**JNJ-7706621**	**tool**	**na**	**oncology**	3.73	3.73		
**PI-103**	**tool**	**na**	**oncology**	0.05	0.05		
